# Establishment of primary cell cultures from canine mammary gland malignant tumours: a preliminary study

**DOI:** 10.2478/jvetres-2025-0007

**Published:** 2025-03-01

**Authors:** Patŕicia Petrouškova, Nikola Hudáková, Viera Almášiová, Alexandra Valenčáková, L’ubica Horňáková, Mykhailo Huniadi, Daša Čížková

**Affiliations:** Department of Epizootiology, Parasitology and Protection of One Health, University of Veterinary Medicine and Pharmacy in Košice, 041 81 Košice, Slovak Republic; Small Animal Clinic, University Veterinary Hospital, University of Veterinary Medicine and Pharmacy in Košice, 041 81 Košice, Slovak Republic; Department of Morphological Disciplines, University of Veterinary Medicine and Pharmacy in Košice, 041 81 Košice, Slovak Republic; Institute of Neuroimmunology, Slovak Academy of Sciences v.v.i., 845 10 Bratislava 45, Slovak Republic

**Keywords:** canine mammary gland tumour, primary cell culture, MUC1, CK8/18, Ki-67

## Abstract

**Introduction:**

Canine mammary gland cancer (CMGC) is the most common neoplastic condition in bitches and is often fatal. There are limited treatment options for CMGC. Primary cell cultures from mammary tumours are promising preclinical *in vitro* models in which to study personalised treatment approaches. This preliminary study aimed to establish primary cell cultures from two canine mammary gland neoplasms: a common solid adenocarcinoma and a rare carcinosarcoma.

**Material and Methods:**

Tumour masses were collected from a 13-year-old and a 16-year-old German shepherd. Tumour cells were isolated by mechanical disaggregation and enzymatic digestion of masses with 0.05% type IV collagenase. Primary cell cultures were validated by immunocytochemistry for specific markers including mucin 1 (MUC1), cytokeratin 8 and 18 (CK8/18) and Kiel 67 (Ki-67).

**Results:**

Primary cell cultures achieved confluency by day 7 of culture, displaying polygonal cellular morphology. Cultures of both cell types exhibited strong positivity for MUC1 of >99% and high Ki-67 proliferation activity of 43.1% ± 0.5% in the solid adenocarcinoma-derived positive cells and 87.9% ± 2.7% in the carcinosarcoma-derived positive cells. Positivity was observed for CK8/18 of 98.1% ± 0.3% in cells derived from solid adenocarcinoma and 31.6% ± 1.5% in cells derived from carcinosarcoma.

**Conclusion:**

With further characterisation, the primary cell cultures established in this study can be expected to show considerable potential as foundational *in vitro* models for cancer research.

## Introduction

Canine malignancies represent a significant clinical problem in veterinary medicine. Epidemiological data reveal that cancer affects approximately one in every four dogs during their lifetime and kills nearly half of all dogs aged over 10 (34). Canine mammary gland cancer (CMGC) is the second most diagnosed type of cancer in dogs. It constitutes the predominant neoplastic disease in bitches, comprising over 50–70% of all tumour occurrences (33). Risk factors associated with the more frequent development of CMGC include breed (*e.g*. Labrador retrievers, cocker spaniels, Irish setters, German shepherds, miniature or toy poodles being at higher risk), advanced age (typically >8 years), hormonal status (high levels of oestrogen and progesterone being dangerous) and being overweight (4).

Despite improved and more accessible veterinary care, treating mammary tumours always presents a substantial challenge for veterinarians. Therapeutic outcomes for dogs with mammary cancer are poor, primarily due to the reduced effectiveness of chemotherapy in dogs. The gold standard treatment regimen typically involves radical mastectomy combined with chemotherapy. Currently, there is no standardised chemotherapy protocol for bitches, and chemotherapy has demonstrated limited efficacy in prolonging patient survival (35). Only two chemotherapy drugs have eradicated tumours completely (*i.e*. gemcitabine and carboplatin), while many of these drugs are associated with various levels of toxicity (10).

Culturing tumour cells *in vitro* furnishes a valuable preclinical model for studying carcinogenesis processes (such as proliferation, migration and apoptosis), discovering new therapeutics, elucidating drug mechanisms and identifying genes implicated in carcinogenesis. Several canine mammary tumour cell lines derived from primary and metastatic tumours have been established in the past few years (6, 7, 11, 17, 18, 22). While cell lines have long been integral to cancer research, a more pressing need has prompted a growing interest in establishing primary cancer cell cultures in recent years. Primary cell cultures are indispensable in cancer research because they provide more accurate and comprehensive *in vitro* models of original tumours, retaining their genetic heterogeneity and phenotypic characteristics and thereby enhancing the development of effective cancer therapies. Primary cell cultures are also essential for advancing personalised medicine, which represents cancer treatment in its improved future form (30). Furthermore, the spontaneous occurrence of neoplasms in bitches and their resemblance to human breast cancer in terms of histological classification, epidemiological and clinical features, molecular targets and biological behaviour suggest that canine mammary gland tumours (CMTs) provide a realistic, diverse and ethically acceptable model for translational research and therapeutic development in human breast cancer (1).

Biomarkers, generally used to determine the diagnosis, prognosis or staging of cancer in both human and veterinary oncology (14), play a crucial role in immunocytochemistry (ICC), where they enable the identification of malignant cells, confirmation of tumour origin and the assessment of tumour aggressiveness. In the context of CMTs, biomarkers such as mucin 1 (MUC1), cytokeratins 8 and 18 (CK8/18) and Kiel 67 (Ki-67) are essential for confirming the epithelial origin of the tumour cells and evaluating their proliferative potential (15).

In this preliminary study, we established primary cell cultures from two distinct mammary gland malignant tumours: a common solid adenocarcinoma and an uncommon carcinosarcoma. To the best of our knowledge, this is the first culture of mammary gland carcinosarcoma. After further characterisation, these primary cell cultures will have great potential for advancing our understanding of mammary cancer biology, molecular processes and patient-specific therapy options.

## Material and Methods

### Ethics statement

The study was performed with the approval of the Ethics Committee of the University of Veterinary Medicine and Pharmacy in Košice in the Slovak Republic (EKVP/2023-02). Informed consent was obtained from the owners of the dogs.

### Collection of tumour samples

An intact 13-year-old and an intact 16-year-old German shepherd bitch were diagnosed at the Small Animal Clinic at the University Veterinary Hospital of the University of Veterinary Medicine and Pharmacy in Košice. Palpation of the mammary gland, ultrasonographic examination of the abdomen for detection of possible metastases in internal organs and lymph nodes and radiograph examination of the chest for detection of lung metastases confirmed CMGC.

Tumour masses were surgically excised during mastectomy in sterile conditions following standard operating procedures. The tumour in the 13-year-old dog (Tu13Y) was solid and irregular, with a size of 3 × 4 cm, and was located in the second mammary gland on the left side. The tumour in the 16-year-old dog (Tu16Y) exhibited a solid-elastic consistency, measured 7 × 9 cm, and was located in the fourth mammary gland on the right side. To ensure consistent histopathological analyses, three 1 cm^3^ specimens from different parts of Tu13Y and six specimens of the same size from different parts of Tu16Y were processed *via* routine formalin fixation, paraffin embedding and haematoxylin-eosin staining. Identification of the histological subtype was performed by two independent pathologists according to World Health Organization criteria (23). For cell culture preparation, tumour specimens of approximately 1 cm^3^ were taken from the same parts of the tumours as the specimens used for histopathological examination and placed in phosphate-buffered saline (PBS; Sigma-Aldrich, St. Louis, MO, USA) containing 2% penicillin-streptomycin-amphotericin B solution (ATB/ATM; Biowest, Nuaillé, France) and 0.02% gentamicin solution (50 mg/mL; Sigma-Aldrich).

### Isolation and cultivation of canine mammary tumour cells

Tumour cells were isolated as previously described (17) with minor modifications. Briefly, cancerous mammary tumour tissues were cut into small pieces (<0.5 mm) and washed 3 times with PBS (Sigma) containing 1% ATB/ATM (Biowest) and 0.02% gentamicin (Sigma-Aldrich). Tumour fragments were enzymatically dissociated with 0.05% Collagenase Type IV (Gibco, Grand Island, NY, USA) in 5 mL of Earle’s balanced salts solution (EBSS) without calcium and magnesium (Biowest) containing 1% ATB/ATM (Biowest) for 2 h at 37°C in a humidified atmosphere containing 5% CO_2_. The disaggregated tissue suspensions were separated using a 75-μm cell strainer (Corning, Corning, NY, USA) into a 50 mL centrifuge tube. The suspensions were centrifuged at 1,200 rpm for 5 min at room temperature (RT) and the pellets were resuspended in EBSS. The cell suspensions were subsequently centrifuged again at 1,200 rpm for 5 min at RT, and this step needed to be repeated three times to remove excess collagenase and to complete the isolation of tumour cells. Finally, isolated cell pellets were resuspended in high-glucose Dulbecco’s modified Eagle’s medium (Sigma-Aldrich) supplemented with 10% foetal bovine serum (FBS) (Gibco), 1% L-glutamine (Sigma-Aldrich), 1% ATB/ATM (Biowest), and 0.01% gentamicin solution (Sigma-Aldrich).

The cells were added into 25 cm^2^ culture flasks (TPP Techno Plastic Products, Trasadingen, Switzerland) and cultivated at 37°C and 5% CO_2_. Cell debris and non-adherent cells were removed after 24 h of cultivation by washing the cells with Dulbecco’s PBS (DPBS; Sigma-Aldrich). The cell culture medium was changed to fresh every 48 to 96 h. Cell growth was monitored daily by inverted microscopy (Zeiss Axiovert 200, Zeiss, Oberkochen, Germany).

When the cell cultures reached 90% confluence, the cells were washed with DPBS (Sigma-Aldrich) and detached using 0.25% trypsin (Gibco) containing ethylenediaminetetraacetic acid (EDTA) at 37°C and 5% CO_2_. Trypsinisation was terminated using FBS (Gibco) at a ratio of 1 : 1 (v/v). The resultant cell suspensions were centrifuged at 1,250 rpm for 10 min at RT. Cell pellets were dissociated in 1 mL of culture medium. Secondary cultures were obtained by placing the digested cells in a 24-well culture plate (TPP) at a density of ~1×10^4^ cells/mL on collagen-coated coverslips (details are in the ICC section). The rest of the trypsinised cells were cryopreserved.

### Immunocytochemistry

The ICC assay was performed to detect the expression of selected CMT markers (MUC1, CK8/18 and Ki-67). The cells in an approximate amount of 1×10^4^/mL were cultured at 37°C and 5% CO_2_ in a 24-well plate (TPP) on coverslips (12 mm) coated with Collagen, Type I (Sigma-Aldrich). Once the confluency reached 70%, the cells were washed with DPBS (Sigma-Aldrich) and fixed in 4% paraformaldehyde (Sigma-Aldrich) in PBS for 15 min at RT. The cells were then permeabilised with 0.2% Triton X-100 in PBS (0.2% PBS-T) for 20 min at RT and blocked for 1 h at RT using 10% normal goat serum (Sigma-Aldrich) in 0.2% PBS-T. After blocking, cells were exposed to primary antibodies (Table 1) and incubated at 4°C overnight. The next day, cells were washed with 0.05% PBS-T for 5 min at RT with constant shaking and incubated with secondary antibodies (Table 1) for 1 h at RT with gentle shaking. The cells were washed again three times for 5 min with 0.05% PBS-T and once with PBS. Finally, coverslips were mounted using a Fluoroshield Histology Mounting Medium with DAPI (4′,6-diamidino-2-phenylindole) (Sigma-Aldrich) and observed through fluorescence microscopy (LSM-710, Zeiss). The ICC was performed in triplicate per primary antibody for both CMTs.

**Table 1.** Specific primary and secondary antibodies used for immunocytochemistry

Primary antibodies							
Marker	Primary antibody	Type, clone	Host, isotype	Reactivity	Dilution	Catalogue No.	Supplier
mucin 1	MUC1	mAb, MUC1/955	mouse, IgG	human, mouse	1 : 150	NBP2-44658	Novus Biologicals, Centennial, CO, USA
cytokeratin 8 cytokeratin 18	Cytokeratins 8/18	mAb, 5D3	mouse, IgG	human, mouse	1 : 100	NB120-17139	Novus Biologicals
Ki-67	Anti-Ki-67	pAb, N/A	rabbit, IgG	human, mouse	1 : 300	ab15580	Abcam, Cambridge, UK

Secondary antibodies							
Primary antibody	Secondary antibody	Type	Host, isotype	Reactivity	Dilution	Catalogue No.	Supplier
MUC1	Alexa Fluor 488 goat anti-mouse IgG (H+L)	pAb	goat, IgG	mouse	1 : 300	A-11001	Invitrogen, Carlsbad, CA, USA
Cytokeratins 8/18	Alexa Fluor 594 goat anti-mouse IgG (H+L)	pAb	goat, IgG	mouse	1 : 300	A-11005	Invitrogen
Anti-Ki-67	Alexa Fluor 594 goat anti-rabbit IgG (H+L)	pAb	goat, IgG	rabbit	1 : 300	A-11037	Invitrogen

1 mAb – monoclonal antibody; pAb – polyclonal antibody; MUC1 – mucin 1; Ki-67 – Kiel 67; N/A – not available

To confirm the specificity of primary antibodies, non-cancerous cells isolated from healthy canine mammary glands exhibiting certain patterns of MUC1 (9) and CK8/18 (28) and no or minimal Ki-67 (29) expression were used. This canine mammary gland tissue was obtained during necropsy examination of a female dog with normal non-neoplastic mammary glands. The cells were isolated as described above. For negative controls, primary antibodies were excluded from the protocol.

### Immunocytochemistry interpretation

Expression of CK8/18 and Ki-67 was assessed based on the number of marker-positive cells among a minimum of 500 neoplastic cells (nuclei) (25) per replicate in randomly chosen fields (×100). The number of cells was determined by manual image analysis with the help of the Cell Counter ImageJ plugin v3.0.0 (32). The cells were considered strongly positive for MUC1 at the threshold ≤50% (21), and positive for CK8/18 at the threshold ≤10% (13). For Ki-67, the >33.3% threshold for highly proliferative positive cells was used (25). Tumour cell nuclei displaying granular staining were classified as positively stained, regardless of intensity. Any cytoplasmic immunoreactivity was considered non-specific and therefore not taken into consideration. The standard deviation was expressed as the mean (±) percentage of marker-positive cells from each replicate.

## Results

### Establishing primary canine mammary cancer cell cultures

**Fig. 1.**
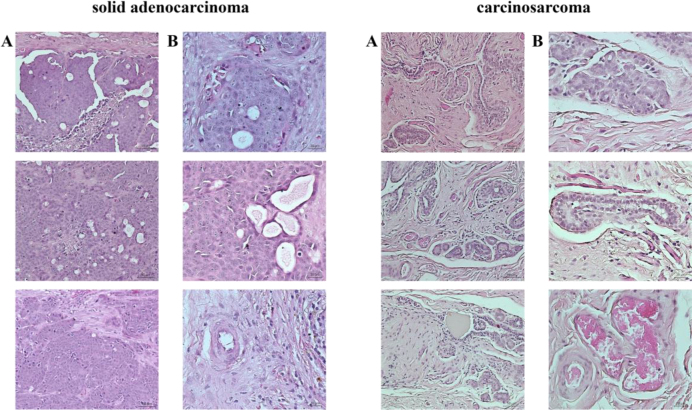
Microphotographs of mammary gland tumours. A – magnification 200×, scale bar 50 μm; B – magnification 400×, scale bar 20 μm

Histopathological evaluation of the 13-year-old German shepherd’s tumour sample revealed a solid adenocarcinoma, and this procedure on the 16-year-old dog’s neoplastic tissue showed it to be a carcinosarcoma (Fig. 1).

Employing the protocol outlined in this study, the primary canine mammary cell cultures of solid adenocarcinoma and carcinosarcoma were successfully established.

**Fig. 2.**
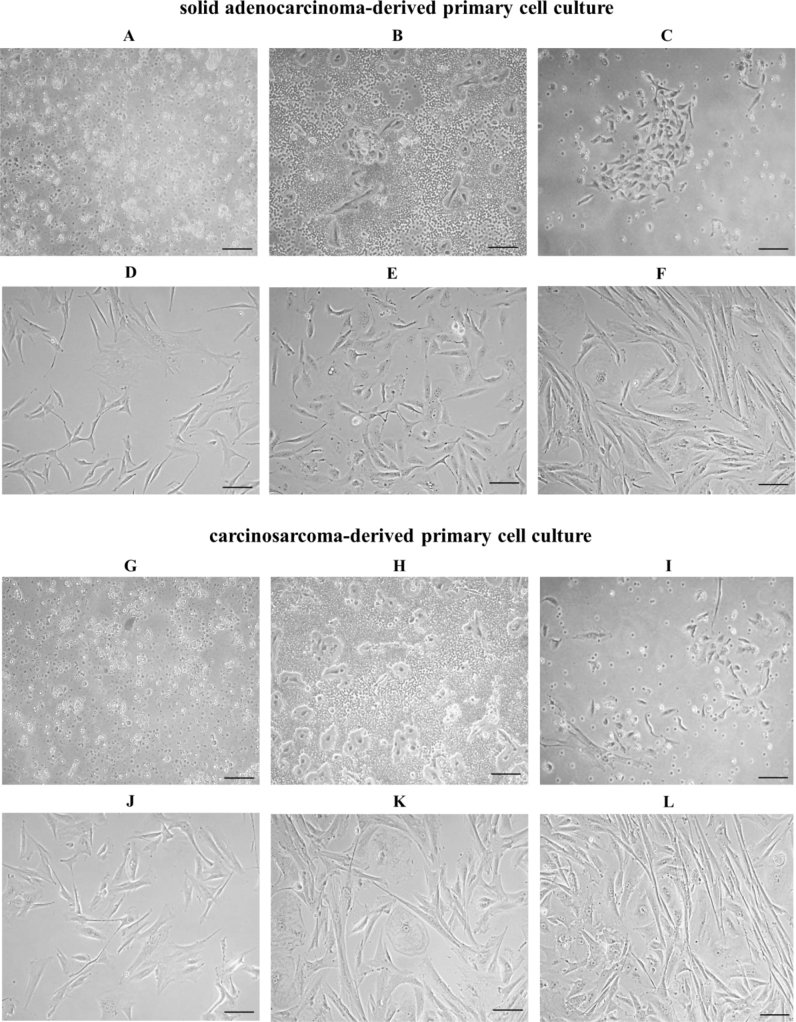
Primary cell cultures derived from mammary gland solid adenocarcinoma and carcinosarcoma. A and G – cellular debris and blood cells present directly after plating the cells on day *in vitro* (DIV) 0; B and H – cells before washing on DIV 1; C and I – cells after washing on DIV 1. Washing the cells effectively removed the majority of cellular debris and non-adherent cells; D and J – enlarged irregular and star-shaped cells on DIV 3; E and K – larger polygonal and irregularly shaped cells on DIV 5; F and L – cells with a range of morphologies from spindle-shaped to polygonal-shaped on DIV 7. Magnification 200×, scale bar 50 μm

Cells isolated following tumour fragmentation and enzymatic digestion exhibited a round-to-oval morphology upon plating (day *in vitro* 0 – DIV 0). Additionally, a significant presence of blood cells and cellular debris was noted (Fig. 2A and G). On the following day after isolation (DIV 1), some adhesion of tumour cells to the surface of the culture flask was observed (Fig. 2B and H). Cell debris and non-adherent cells were removed by washing the cells, which left small bipolar cells predominant (Fig. 2C and I). On the third day of cultivation (DIV 3), the cells reached ~50% confluency. In addition to spindle-shaped cells, the presence of enlarged irregular and star-shaped cells was observed. Cells formed elongated protrusions facilitating intercellular connections (Fig. 2D and J). More significant changes in cell morphology were observed by the fifth day of cultivation (DIV 5). With cellular confluency reaching approximately 70%, a predominance of larger polygonal and irregularly shaped cells emerged (Fig. 2E and K). By the seventh day of cultivation (DIV 7), a confluence of ~90–95% was achieved, with cells exhibiting a range of morphologies from spindle-shaped to irregularly shaped (Fig. 2F and L).

### Immunocytochemical characterisation of canine mammary cancer cells

**Fig. 3.**
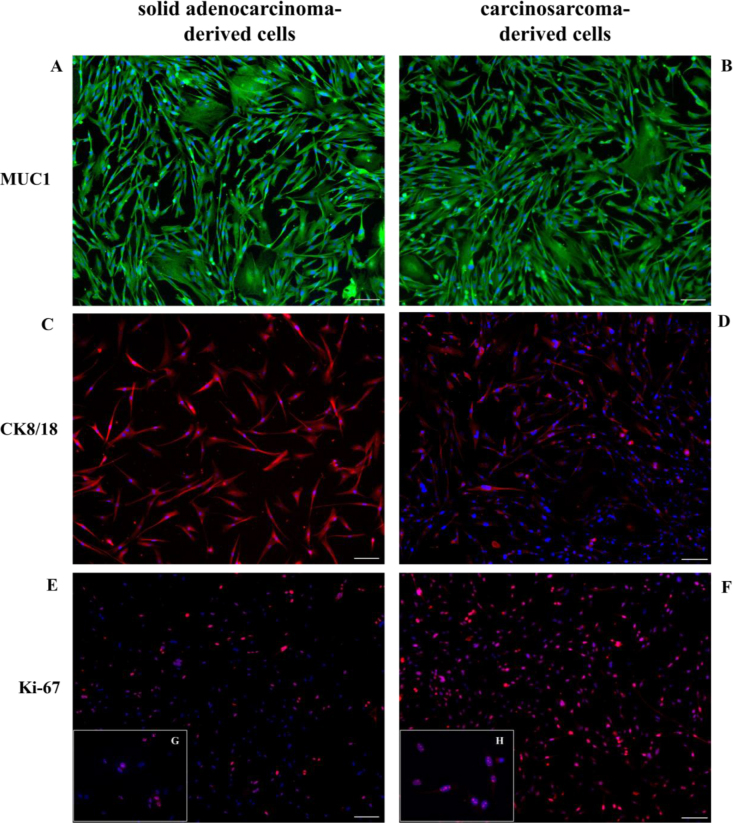
Immunofluorescence staining to show mucin 1 (MUC1), cytokeratins 8 and 18 (CK8/18) and Kiel 67 (Ki-67) expression in canine mammary gland tumour cells. A and B – solid adenocarcinoma-derived and carcinosarcoma-derived cells expressing MUC1; C – solid adenocarcinoma-derived cells showing higher expression of CK8/18 than carcinosarcoma-derived cells; D – carcinosarcoma-derived cells showing lower expression of CK8/18 than solid adenocarcinoma-derived cells; E – solid adenocarcinoma-derived cells showing lower expression of Ki-67 than carcinosarcoma-derived cells; F – carcinosarcoma-derived cells showing higher expression of Ki-67 than solid adenocarcinoma-derived cells; G and H – 400× magnification of details of E and F. Nuclei were stained with 4′,6-diamidino-2-phenylindole. Magnification 100×, scale bar 100 μm

**Fig. 4.**
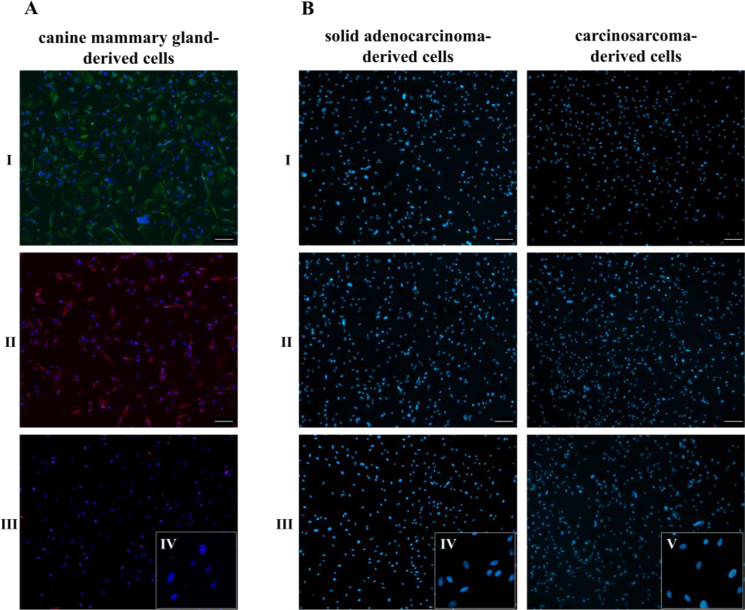
Immunocytochemistry controls verifying the specificity of primary antibody staining. A – non-cancerous cells isolated from canine mammary glands demonstrating weak positive staining for mucin 1 (MUC1) (I) and cytokeratins 8 and 18 (CK8/18) (II), and minimal Kiel 67 (Ki-67) staining (III). (IV) is a 400× magnification of details of (III); B – negative controls where primary antibodies (MUC1 (I), CK8/18 (II) and Ki-67 (III)) were excluded from the assay. Nuclei were stained with 4′,6-diamidino-2-phenylindole. Magnification ×100, scale bar 100 μm

Expression of MUC1, CK8/18 and Ki-67 was detected through ICC (Fig. 3). Tumour cells exhibited significantly higher marker positivity compared to healthy mammary gland cells (Fig. 4A). Strong MUC1 expression was observed in 99.1% ± 0.3% and 99.2% ± 0.2% of solid adenocarcinoma-derived cells and carcinosarcoma-derived cells, respectively (Fig. 3A and B). Cytokeratins 8 and 18 were detected in both cultures, 98.1% ± 0.3% of solid adenocarcinoma-derived cells and 31.6% ± 1.5% of carcinosarcoma-derived cells being positive for them (Fig. 3C and D). Kiel 67 expression indicated high proliferative activity, as 43.1% ± 0.5% of solid adenocarcinoma-derived cells (Fig. 3E and G) and 87.9% ± 2.7% of carcinosarcoma-derived cells (Fig. 3F and H) were positive. The control assays showed no non-specific staining (Fig. 4B).

## Discussion

Canine mammary gland tumours, which are the most prevalent among unspayed bitches, represent up to 70% of diagnoses in veterinary oncology. The annual incidence is around 200 cases per 100,000 dogs, and over 50% of them are malignant (33). This makes CMGC a significant health concern in veterinary medicine. Given the high prevalence and clinical importance of CMTs, there is a growing need for reliable *in vitro* models by which to better understand their complex biology and more effectively develop targeted therapies. In this preliminary study, we successfully established primary cell cultures from malignant CMTs histologically diagnosed as a solid adenocarcinoma and a carcinosarcoma. These cultures provided a valuable *in vitro* model to study tumour behaviour, treatment responses and molecular characteristics specific to each patient’s CMTs. Although limited in scope, this work takes an essential step towards creating more robust models, such as stable cell lines, for future research in veterinary and comparative oncology and contributes to the growing body of knowledge on CMTs.

Primary cancer cell cultures serve as valuable *in vitro* preclinical models in oncological research. Being derived directly from tumour tissues of individual animals, these cell cultures can be used to plan personalised treatment approaches tailored to each patient’s genetic and molecular characteristics. Thus, cell cultures are of great significance to understanding the development of CMGC. They enable the creation of sophisticated models to elucidate the intricate processes underlying tumorigenesis and explain the biological properties inherent to tumour cells. Moreover, canine mammary tumours exhibit biological behaviours and molecular characteristics similar to human breast cancer. These advantages, together with their spontaneous occurrence needing no chemical induction or genetic modification, render CMTs an ideal translational model for studying human breast cancer (1).

Tumour tissues were obtained from two unspayed German shepherd female dogs, a breed known for its higher susceptibility to mammary gland neoplasia (8). The protocol employed in this study is a minor modification of a previously described procedure (17) and employed a combination of mechanical dissociation and enzymatic digestion. The mechanical dissociation involved cutting the tissue into smaller pieces, thereby enhancing accessibility for enzymatic digestion. Fragments of tumour tissue were initially rinsed in PBS with antibiotics as described in other studies (18, 22, 36). An alternative approach involved washing the tissue in a culture medium supplemented with antibiotics (6). For enzymatic digestion, we employed 0.05% type IV collagenase. This type of collagenase was selected based on the protocol of Lainetti *et al*. (17), which demonstrated superior cell isolation outcomes than were obtained with types I and II collagenase. However, other studies have demonstrated the successful isolation of tumour cells with the use of type IA (6) or type II collagenase (22, 36), 0.25% trypsin/EDTA (18) or TrypLE Express trypsin-like enzyme (7). A 2-h digestion period of the tissue samples proved sufficient for the isolation of tumour cells with no observable cell damage and satisfactory culture expansion. This duration was notably shorter than the digestion periods reported in other studies, where the process was extended to 4 h (17, 22), 6 h (7) or overnight (36). The established cell cultures exhibited a relatively rapid growth rate, with a monolayer formation observed by day 7 post isolation. This rapid proliferation indicated that the isolated tumour cells adapted well to the *in vitro* conditions and displayed robust growth characteristics early in culture. Achieving a monolayer within this timeframe was consistent with the expected behaviour of aggressive tumour cells, which tend to exhibit high proliferative capacity. This growth rate not only underscored the efficiency of the isolation method used but also suggested that the cultured cells retained their tumorigenic properties, making them suitable for further biological and molecular analyses.

Canine mammary gland tumours are extensively characterised by molecular biomarkers which help in understanding tumour biology, progression and potential treatment targets. Commonly investigated biomarkers in CMTs include hormone receptors such as oestrogen and progesterone receptors, which are used to assess tumour responsiveness to hormonal therapies, as well as human epidermal growth factor receptor 2, a receptor associated with more aggressive tumour behaviour. Other markers like p53 (a tumour suppressor protein) and Ki-67 (a proliferation index marker) are frequently used to analyse tumour progression and aggressiveness. Additionally, markers related to their role in immune evasion and tumour invasiveness, such as MUC1, and markers confirming epithelial origin, such as cytokeratins, have gained importance in understanding tumour invasion and metastasis (15). Immunocytochemistry plays a crucial role in detecting these biomarkers at the cellular level and makes the precise characterisation of tumour cells possible *in vitro*. In this study, we selected MUC1, CK8/18 and Ki-67 as our primary biomarkers to confirm the tumorigenic nature of the cell cultures. Mucin 1 was selected because it is overexpressed in various epithelial cancers, including CMTs, where it plays a key role in tumorigenesis and metastatic processes (9). Both cell cultures were highly positive for MUC1. Also known as the cluster of differentiation 227, cancer antigen 15–3 or Krebs von den Lungen-6, MUC1 is a transmembrane glycoprotein involved in protecting the epithelial barrier or cellular adhesion (12). Although MUC1 as a transmembrane glycoprotein is typically expressed in the cell membrane of healthy tissue, during malignant transformation it can be overexpressed both on the cell surface and within the cytoplasm of tumour cells (9). During the malignant process, MUC1 functions as an anti-adhesive molecule, thereby increasing the metastatic and invasive potential of tumour cells (21). Increased expression of MUC1 in malignant mammary gland tumours (carcinomas and carcinosarcomas) compared to its expression in benign mammary gland tumours and healthy gland tissue has also been observed in other studies (9, 20, 24). Several studies have also shown that overexpression of MUC1 is closely associated with tumour malignancy and poor prognosis, especially in carcinomas (20, 37). Consistent with these findings, elevated MUC1 expression in tumour cells and minimal expression in healthy mammary gland cells were also our observations.

Cytokeratins 8 and 18 were employed to confirm the epithelial origin of the cultured cells, which is particularly useful in distinguishing between different histological subtypes of CMT, and to exclude fibroblast contamination. A major subgroup of intermediate filaments, CKs are specific to epithelial cells, with CKs 8, 18 and 19 being the most abundant. In normal canine mammary glands, the epithelial cells express CKs (28). In malignant CMTs, CKs are typically overexpressed in carcinomas and serve as highly valuable tumour markers in oncology (3); consistent CK18 expression has been observed in canine mammary gland carcinomas (17, 24, 31). We observed strong CK8/18 staining of solid adenocarcinoma cells. This contrasted with the moderate expression of CK8/18 among carcinosarcoma cells, which did not become manifest in approximately 70% of them. This difference was attributed to the microscopic structure of these tumours, as adenocarcinomas are predominantly composed of epithelial cells, whereas carcinosarcomas have a biphasic nature with epithelial and mesenchymal components (2). Staining of CK8/18 highlighted the epithelial elements within carcinosarcoma and confirmed the use of CKs as markers in these mixed malignancies. Since CKs are well-known as markers of epithelial cells, positive staining of both cell cultures serves as a reliable indicator for the absence of fibroblast contamination. Fibroblast overgrowth is a frequent challenge in primary cell cultures derived from tissues, particularly in canine mammary gland tumours, which are predominantly of epithelial origin. Several studies have confirmed the lack of CK expression in cancer-associated fibroblasts, further supporting the use of CK staining to distinguish between epithelial cells and fibroblasts in cell cultures (16, 19, 38).

Finally, Ki-67 was included as the most studied proliferation marker, given its strong association with active cell division. Its expression provides insights into how proliferative a tumour is (27). As a nuclear protein, Ki-67 is detectable only within the cell nucleus of actively proliferating cells during interphase and mitosis (the S and G2 phases) (27). The expression of Ki-67 is influenced by various factors, including tumour size or lymph node metastasis. Several studies have indicated a positive correlation between high Ki-67 expression rate and tumour grade, metastasis, poor prognosis, and low overall survival rate in dogs (26, 27, 39). Regarding mammary gland cancer, different expression rates of Ki-67 in various histotypes of canine mammary carcinomas (5, 31) and adenocarcinomas (22, 26) were observed. With a determined cutoff of 33.3% Ki-67-positive nuclei to separate high versus low proliferative tumours (25), both primary cultures were classified as highly proliferative. However, carcinosarcoma cells were characterised by a twofold higher expression of Ki-67 (87.9% ± 2.7%) than solid adenocarcinoma cells (43.1% ± 0.5%). Generally, higher Ki-67 expression in carcinosarcoma may be explained primarily by carcinosarcoma’s more aggressive nature, higher potential for rapid growth and proliferation activity, local invasion, metastasis and biphasic histology compared to adenocarcinoma, which is composed primarily of malignant epithelial cells (2).

Primary cell cultures offer a unique opportunity to establish cell lines that can be systematically preserved within cell banks. This ensures greater reproducibility of experimental outcomes and enhances the reliability of result validation. For these cell lines from the cultures established in this preliminary study to become lines established as valid research material, they must meet certain criteria, including having altered cell cytomorphology, an enhanced growth rate, increased clonogenicity, reduced serum dependence, ploidy alteration, tumorigenic potential and the ability to undergo unlimited replication cycles (1). Therefore, further testing and comprehensive characterisation are required for the cell cultures established in this attempt.

## Conclusion

In this study, we successfully established two canine cell cultures derived from primary canine mammary gland tumours, specifically solid adenocarcinoma and carcinosarcoma. Both cell cultures exhibited marker characteristics consistent with mammary gland cancer. We believe that with further testing, these cell cultures will be shown to be a valuable cellular model with significant research implications for investigating aspects such as the tumour microenvironment, drug resistance mechanisms and potential therapeutic strategies for mammary gland cancer.
